# Embryonic Stem Cell-Derived L1 Overexpressing Neural Aggregates Enhance Recovery after Spinal Cord Injury in Mice

**DOI:** 10.1371/journal.pone.0017126

**Published:** 2011-03-18

**Authors:** Yi-Fang Cui, Jin-Chong Xu, Gunnar Hargus, Igor Jakovcevski, Melitta Schachner, Christian Bernreuther

**Affiliations:** 1 Zentrum für Molekulare Neurobiologie Hamburg, Universitätskrankenhaus Hamburg-Eppendorf, Universität Hamburg, Hamburg, Germany; 2 Clinical Neurobiology Laboratory, German Primate Center, Leibniz Institute for Primate Research, Göttingen, Germany; 3 W. M. Keck Center for Collaborative Neuroscience and Department of Cell Biology and Neuroscience, Rutgers University, New Jersey, United States of America; 4 Institute of Neuropathology, University Medical Center Hamburg-Eppendorf, Hamburg, Germany; National Institute on Aging Intramural Research Program, United States of America

## Abstract

An obstacle to early stem cell transplantation into the acutely injured spinal cord is poor survival of transplanted cells. Transplantation of embryonic stem cells as substrate adherent embryonic stem cell-derived neural aggregates (SENAs) consisting mainly of neurons and radial glial cells has been shown to enhance survival of grafted cells in the injured mouse brain. In the attempt to promote the beneficial function of these SENAs, murine embryonic stem cells constitutively overexpressing the neural cell adhesion molecule L1 which favors axonal growth and survival of grafted and imperiled cells in the inhibitory environment of the adult mammalian central nervous system were differentiated into SENAs and transplanted into the spinal cord three days after compression lesion. Mice transplanted with L1 overexpressing SENAs showed improved locomotor function when compared to mice injected with wild-type SENAs. L1 overexpressing SENAs showed an increased number of surviving cells, enhanced neuronal differentiation and reduced glial differentiation after transplantation when compared to SENAs not engineered to overexpress L1. Furthermore, L1 overexpressing SENAs rescued imperiled host motoneurons and parvalbumin-positive interneurons and increased numbers of catecholaminergic nerve fibers distal to the lesion. In addition to encouraging the use of embryonic stem cells for early therapy after spinal cord injury L1 overexpression in the microenvironment of the lesioned spinal cord is a novel finding in its functions that would make it more attractive for pre-clinical studies in spinal cord regeneration and most likely other diseases of the nervous system.

## Introduction

Spinal cord injury results in a change, either temporary or permanent, in its motor, sensory, or autonomic functions. Due to cellular loss and an inhibitory tissue environment, regeneration after spinal cord injury is limited (for a recent review, see [1]). Current therapeutic approaches to spinal cord injury do not lead to complete functional recovery. Transplantation of stem cells has been shown to successfully replace host neurons, enhance axonal growth, and improve functional recovery in mouse models of spinal cord injury (for reviews see [2], [3], [4]). Embryonic stem (ES) cells are a possible approach to therapy of spinal cord injury. They are pluripotent cells derived from the inner cell mass of the developing blastocyst that can differentiate into derivatives of all three primary germ layers. Thus, increasing attention has been placed on the role of neurally predifferentiated ES cells in repair [5], [6], [7], [8]. As the injured adult spinal cord is a poor tissue environment for cell survival and neuronal differentiation [2], genetic engineering of stem cells is necessary to improve their regenerative potential. To improve the therapeutic features of stem cells, adhesion molecule overexpression represents a viable approach. The neural cell adhesion molecule L1 is a member of the immunoglobulin superfamily [9], [10], [11], [12] that has been shown to promote neurite outgrowth, neuronal migration, and neuronal survival [9], [10], [12], [13], [14], [15], [16]. Injection of adeno-associated virus and Schwann cells encoding L1 into the acutely injured murine spinal cord enhances regeneration and functional recovery [17], [18]. In a previous study, we showed that ES cells overexpressing L1 promote survival of transplanted cells in the injured spinal cord of adult mice compared to non-transfected ES cells [5]. However, survival of grafted cells was poor in this study with only a minority of non-transfected ES cells surviving four weeks after transplantation. Thus, in the present study we combined the beneficial effects of L1 overexpressing ES cells and a prolonged differentiation protocol for ES cells allowing the transplantation of substrate-adherent embryonic stem cell-derived neural aggregates (SENAs) consisting mainly of differentiated neurons and radial glial cells. Dihne et al. [19] previously showed that transplantation of SENAs in a mouse model of Huntington's disease increased survival of transplanted cells with reduced tumor formation when compared to cells differentiated by the 5 stage protocol [20]. Furthermore, L1 overexpressing SENAs have previously been shown to enhance the survival of grafted cells and to rescue endogenous dopaminergic neurons in a mouse model of Parkinson's disease [16]. Based on these findings, a murine ES cell line constitutively expressing L1 at all stages of differentiation [13] was differentiated by the SENA protocol prior to transplantation in this study to combine the beneficial effects of L1 overexpression and the SENA differentiation protocol to overcome the inhibitory environment of the central nervous system and promote functional recovery after spinal cord injury. In this study, we show that L1 overexpressing SENAs survive better after early transplantation into the lesioned spinal cord and enhance locomotor function when compared to wild-type SENAs. Furthermore, L1 overexpressing SENAs rescue endogenous spinal cord interneurons and motoneurons and promote the regrowth of catecholaminergic nerve fibers distal to the lesion site.

## Results

### L1 overexpressing SENAs, but not wild-type SENAs, improve locomotor function after transplantation into the lesioned spinal cord

**Figure 1:**
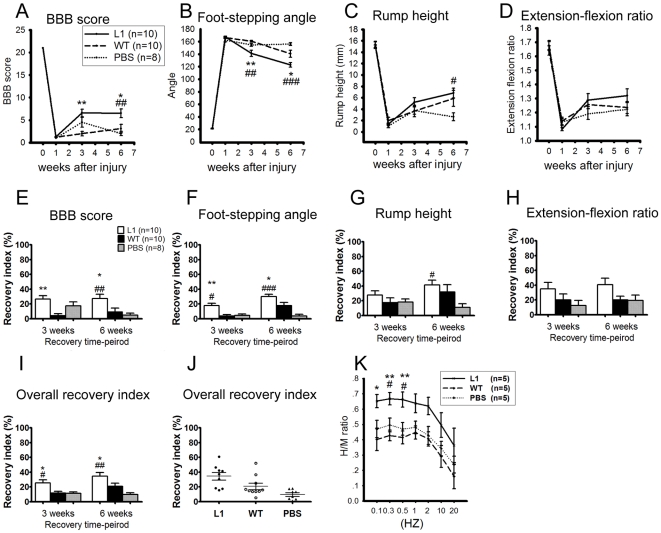
L1 overexpressing SENAs, but not wild-type SENAs, improve locomotor function after transplantation into the lesioned spinal cord. Time course and degree of functional recovery and the H/M ratio after spinal cord compression injury in mice grafted with L1 overexpressing SENAs (n = 10), wild-type (WT, n = 10) SENAs, or sham-injected with PBS (n = 8) is shown. Shown are mean values ± SEM from the BBB scores (**A**), the foot stepping angles (**B**), the rump-height indices (**C**), and the extension-flexion ratio (**D**) one to six weeks after injury. Recovery indices, as shown by mean values ± SEM, were calculated from these raw data (E, F, G, H). In (**I**) the overall recovery index six weeks after injury is shown. Individual values of overall recovery index six weeks after injury are shown in panel (**J**). The numbers of mice studied per group are given in panel **A**. Statistical analysis was performed by Tukey's One-way ANOVA (*, ** *p*<0.05, 0.01 when compared between L1 and wild-type group at a given time point; #, ##, ### *p*<0.05, 0.01, 0.001 when compared to the PBS group at a given time point). (Recovery Index = [(X_7+n_−X_7_)/(X_0_−X_7_)]×100, where X_0_, X_7_ and X_7+n_ are values prior to operation, seven days after injury, and a time-point n days after spinal cord injury, respectively). (**K**) The alterations of M- and H-responses were measured in the plantar muscle during repetitive stimulation of the sciatic nerve with electric pulses at frequencies ranging between 0.1 and 20 Hz. Note the significantly increased H/M ratio in mice grafted with L1 overexpressing SENAs at low stimulation frequencies. Shown are mean values (± SEM) of H/M ratios at different stimulation frequencies at six weeks after spinal cord injury. (*, ** *p*<0.05 and 0.01, when compared to wild-type group, # *p*<0.05, when compared to the PBS group. One-way ANOVA with Tukey's *post hoc* test was performed for statistical evaluation; *n* = 5 mice per group).

To evaluate the effects of the transplantation of SENAs overexpressing the neural cell adhesion molecule L1 into the lesioned spinal cord, L1 overexpressing enhanced green fluorescent protein (EGFP)-positive ES cells and wild-type EGFP-positive ES cells were differentiated according to the SENA differentiation protocol giving rise to SENAs consisting mainly of neurons and radial glial cells. These L1 overexpressing SENAs had previously been shown to enhance the survival of grafted cells and to rescue endogenous dopaminergic neurons in the MPTP-model of Parkinson's disease [16]. In the present study, L1 overexpressing and wild-type SENAs differentiated to day 7 of stage 5, or PBS as a control, were injected rostral and caudal to the center of the lesion three days after compression-lesioning of the spinal cord of C57BL/6J mice. Spinal cord compression injury caused severe locomotor disabilities in all three experimental groups as estimated by the Basso, Beattie, Bresnahan (BBB) score one week after injury (Fig. 1A). Three and six weeks after injury, mice transplanted with L1 overexpressing SENAs showed an increased BBB score when compared to mice grafted with wild-type SENAs or sham-injected with PBS indicating an enhanced locomotor functional recovery (Fig. 1A, E). Besides the BBB score, we analyzed the plantar stepping ability of the animals by measuring the foot-stepping angle [21]. This parameter revealed, in agreement with the BBB scores, enhanced functional recovery in mice grafted with L1 overexpressing SENAs when compared to mice transplanted with wild-type SENAs and PBS three and six weeks after injury (Fig. 1B, F). As the foot-stepping angle is a measure of involuntary movement rather than of more complex motor functions, the rump-height index, a parameter to estimate the ability to support body weight during ground locomotion, was analyzed (Fig. 1C, G). This parameter also indicated enhanced recovery in mice grafted with L1 overexpressing SENAs compared to mice grafted with PBS, but only a tendency towards improved recovery when compared to mice grafted with wild-type SENAs six weeks after transplantation. Moreover, the extension-flexion ratio, a parameter to judge voluntary movements without body weight support, revealed no significant differences among the experimental groups of mice, although L1 overexpressing SENAs showed a tendency towards improved motor function when compared to wild-type SENAs and sham-injected PBS three and six weeks after injury (Fig. 1D, H). From the values of the parameters at different time points shown in Fig. 1E, F, G, H, group mean values were determined (Fig. 1I) and an overall recovery index for each animal was calculated (Fig. 1J) indicating an improved outcome in mice grafted with L1 overexpressing SENAs when compared to mice grafted with wild-type SENAs or sham-injected with PBS.

Thus, transplantation of L1 overexpressing SENAs enhanced functional recovery most prominently at the level of involuntary movements when compared to wild-type SENAs or sham-injected PBS, although a tendency towards enhanced locomotor recovery at the level of voluntary movements was observed in mice after transplantation of L1 overexpressing SENAs that was, however, statistically not significant.

### L1 overexpressing SENAs transplanted into the lesioned murine spinal cord alter stimulated electrophysiological recordings of the sciatic nerve indicating enhanced functional recovery

Lee et al. [22] showed that locomotor function recovery in mice is associated with elevation in the H-reflex. To electrophysiologically evaluate mechanisms underlying the enhanced locomotor function recovery in the mice transplanted with L1 overexpressing SENAs, we analyzed the reflex responses in C57BL/6J mice six weeks after compression injury of the spinal cord. Electrical stimulation of the sciatic nerve led to typical electromyographic responses at baseline stimulation frequencies (0.1 Hz–20 Hz) consisting of a short-latency M-(muscle) wave and a long-latency H-wave elicited by stimulation of motor axons and afferent type Ia fibers, respectively. Based on these results, the H/M ratio was calculated (Fig. 1K). L1 overexpressing SENAs showed an increased H/M ratio at low stimulation frequencies ranging from 0.1 Hz to 0.5 Hz when compared to wild-type SENAs six weeks after transplantation and an increased H/M ratio at frequencies ranging from 0.2 Hz to 0.5 Hz when compared to sham-injected mice indicating enhanced functional recovery in mice grafted with L1 overexpressing SENAs versus wild-type SENAs and sham-injected PBS.

### L1 overexpression in SENAs reduces glial scar formation after transplantation into the lesioned spinal cord

**Figure 2:**
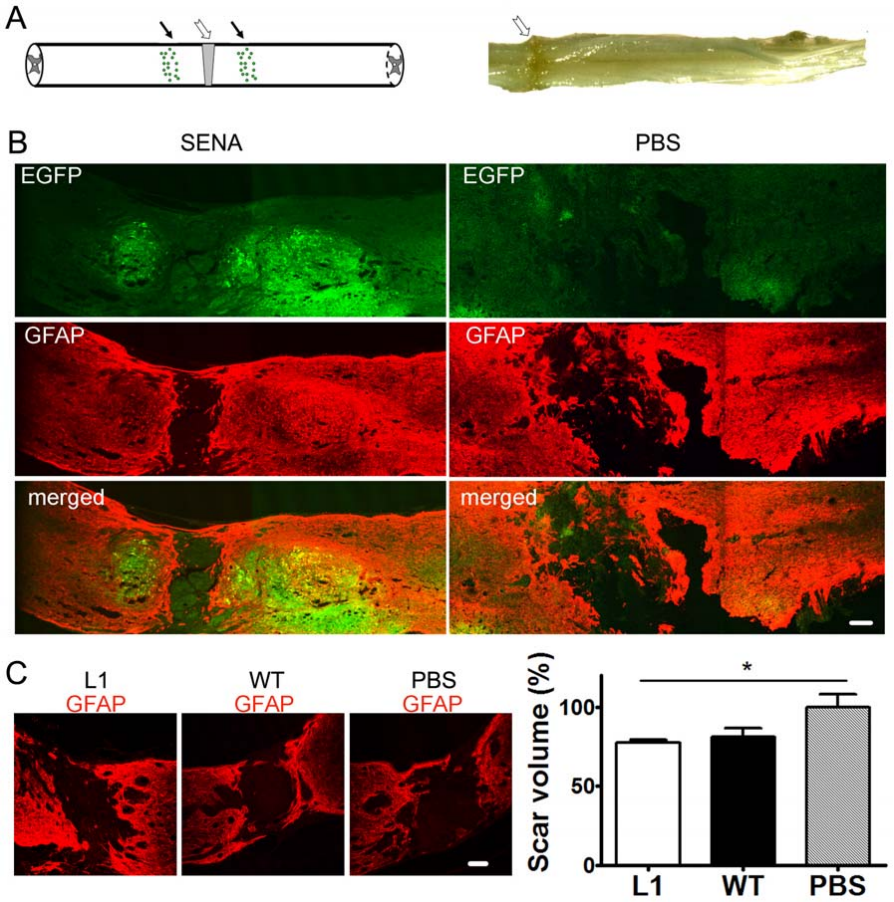
L1 overexpression in SENAs reduces the scar volume after transplantation into the compression-lesioned spinal cord of adult mice. (**A**) Spinal cords were compressed at the T8–T10 level (indicated by a hollow arrow). SENAs were grafted 0.5 mm rostral and caudal to the lesion site (indicated by arrows). (**B**) Localization of SENAs (EGFP-positive) (green) in the lesioned spinal cords six weeks after transplantation. PBS was injected into lesioned spinal cords as control. The lesion site is delineated by glial fibrillary acidic protein (GFAP) expressing astrocytes (red). Scale bar, 100 µm. (**C**) Immunofluorescence of a parasagittal section stained with antibodies against GFAP (red) at the lesion site six weeks after transplantation of L1 overexpressing SENAs (L1), wild-type (WT) SENAs, or sham-injection of PBS. Mean relative scar volumes ± SEM are displayed in the diagram (L1, n = 7; WT, n = 7, PBS, n = 5). The scar volume in the PBS group was adjusted to 100%. Note the decreased scar volume in mice grafted with L1 overexpressing SENAs. One-way ANOVA with Tukey's *post hoc* test was performed for statistical evaluation. (* *p*<0.05). Scale bar, 100 µm.

To elucidate the mechanisms of enhanced locomotor recovery in mice transplanted with L1 overexpressing SENAs, a morphological analysis of graft and host tissue was performed. SENAs were detectable by their green fluorescence at the injection sites (Fig. 2B). Six weeks after transplantation, glial scar formation was compared in mice transplanted with L1 overexpressing or wild-type SENAs and in mice sham-injected with PBS (Fig. 2A, B). The volume of the glial scar correlates with locomotor function in the injured murine spinal cord [21]. Mice grafted with L1 overexpressing SENAs showed a reduced scar volume when compared to sham-injected animals, but only a slightly although not significantly reduced scar volume in comparison with mice grafted with wild-type SENAs (Fig. 2C).

### L1 overexpression in SENAs enhances graft size, number of surviving cells in the graft, and migration from the graft edge and decreases the microglial/macrophage reaction of the host tissue

**Figure 3:**
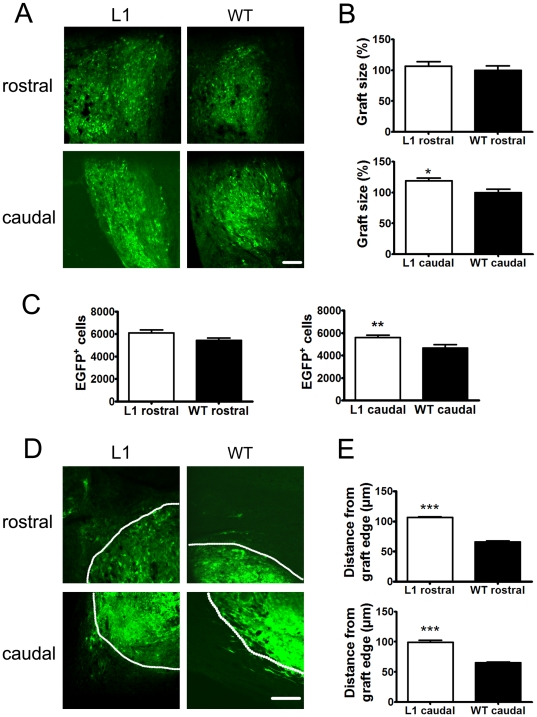
L1 overexpressing SENAs show increased graft size and number of surviving cells as well as enhanced migration from the graft edge. (**A**) Laser scanning microscopy of an L1 overexpressing and a wild-type (WT) SENA graft six weeks after transplantation into the spinal cord rostral and caudal to the lesion site. Grafts were detected by green fluorescence of transplanted cells. Scale bar, 100 µm. Relative graft volume (**B**) and absolute number of EGFP-positive cells (**C**) six weeks after transplantation of L1 overexpressing (n = 7) and WT (n = 7) SENAs rostral and caudal to the lesion site. Shown are mean values ± SEM. The graft volume in the WT group was adjusted to 100%. Student's t test was performed for statistical analysis (*, ** *p*<0.05 and 0.01). (**D**) Laser scanning microscopy of the periphery of L1 overexpressing and wild-type SENA grafts six weeks after transplantation. White line indicates graft edges. Scale bar, 100 µm. (E) Migration distance from the edge of L1 overexpressing (n = 7) and wild-type (n = 7) SENA grafts six weeks after transplantation is shown. (mean values ± SEM). Student's t test was performed for statistical analysis (*** *p*<0.001).

To determine the effects of L1 overexpression on survival of transplanted SENAs and migration of cells from the graft into the host tissue, the number of surviving grafted cells and migration distance of transplanted cells from the graft edge were analyzed six weeks after grafting. L1 overexpressing SENAs showed an increased graft size (Fig. 3A, B) and enhanced numbers of surviving cells (Fig. 3a, c) when compared to wild-type SENAs caudal to the lesion site. Rostral to the lesion site, L1 overexpressing SENAs showed only slightly increased numbers of surviving cells (Fig. 3A, C) and a slightly increased graft size (Fig. 3A, B) when compared to wild-type SENAs. Ki-67 positive cells were rarely found (less than 1%) among grafted cells in both groups indicating the enhanced cell number is not due differences in proliferation between the groups (Fig. S1). Furthermore, migration of grafted cells from the graft edge was enhanced in the grafts both rostral and caudal to the lesion site in L1 overexpressing SENAs versus wild-type SENAs (Fig. 3D, E). Occasionally, migrated cells showed contact with host cells underscoring the importance of migration for the integration of grafted cells (Fig. S2).

**Figure 4:**
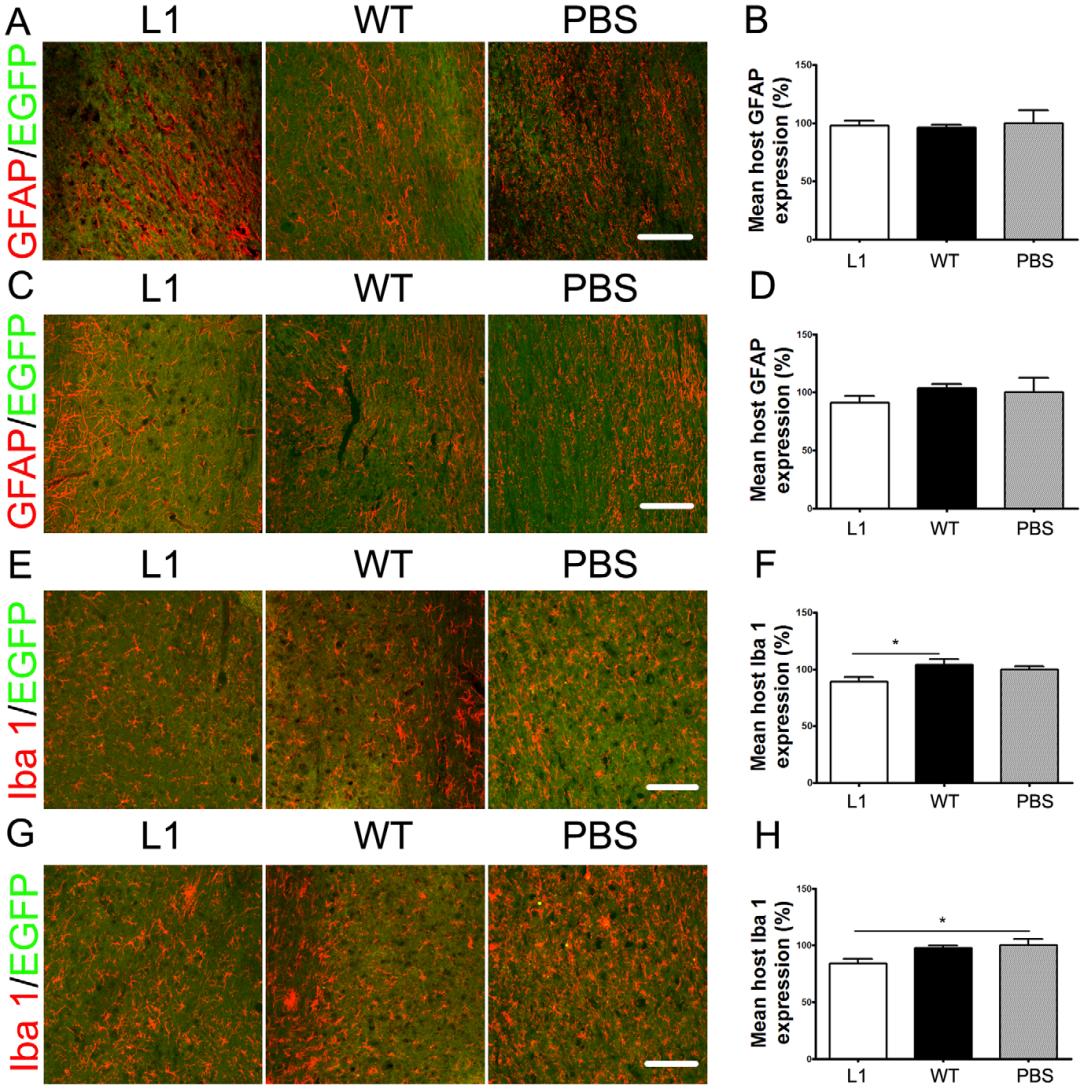
The spinal cords show decreased microglial reaction in mice transplanted with L1 overexpressing SENAs when compared to wild-type SENAs and sham-injected PBS. Confocal images of host spinal cord tissue six weeks after transplantation with L1 overexpressing SENAs, wild-type (WT) SENAs (green), or sham-injected with PBS immunostained with an antibody against the glial fibrillary acidic protein (GFAP, red) rostral (**A**) and caudal (**C**) to the lesion site. Scale bar, 100 µm. Mean fluorescence intensity (± SEM) of GFAP in the host tissue of mice grafted with L1 overexpressing SENAs (n = 5) and WT SENAs (n = 5) rostral (**B**) and caudal (**D**) to the lesion site was compared to the fluorescence intensity in sham-injected animals (n = 5) which was adjusted to 100%. One-way ANOVA with Tukey's *post hoc* test was performed for statistical evaluation. Confocal images of host spinal cord tissue six weeks after transplantation with L1 overexpressing SENAs, wild-type (WT) SENAs (green), or sham-injected with PBS immunostained with an antibody against Iba-1 (red) rostral (**E**) and caudal (**G**) to the lesion site. Scale bar, 100 µm. Mean fluorescence intensity (± SEM) of GFAP in the host tissue of mice grafted with L1 overexpressing SENAs (n = 5) and WT SENAs (n = 5) rostral (**F**) and caudal (**H**) to the lesion site was compared to the fluorescence intensity in sham-injected animals (n = 5) which was adjusted to 100%. One-way ANOVA with Tukey's *post hoc* test was performed for statistical evaluation. (* *p*<0.05).

To analyze whether the increased numbers of surviving cells in L1 overexpressing SENAs were due to differences in the reaction of the host tissue to the graft, reactive astrogliosis and microglia/macrophage reaction of the host tissue surrounding the graft were assessed. No differences were observed between mice transplanted with L1 overexpressing SENAs and wild-type SENAs or sham-injected PBS in the density of glial fibrillary acidic protein (GFAP)-positive astrocytes in the host tissue (Fig. 4A–D). Immunohistochemical analysis with antibodies against ionized binding calcium adapter molecule 1 (Iba-1) (Fig. 4E–H) revealed a decreased density of Iba-1-positive macrophages and/or microglial cells in the tissue surrounding L1 overexpressing grafts when compared to wild-type grafts or sham-injected PBS.

Thus, graft size, cell number, and migration into the host tissue was enhanced in L1 overexpressing versus wild-type SENAs after transplantation caudal to the lesion site. Rostral to the lesion site, migration was enhanced in L1 overexpressing versus wild-type SENAs, while graft size and cell number were only slightly altered. Furthermore, L1 overexpressing SENAs decreased the microglial/macrophage reaction in the host tissue surrounding the graft.

### L1 overexpression in SENAs increases neuronal differentiation and neurite outgrowth of graft-derived neurons and decreases astrocytic differentiation

**Figure 5:**
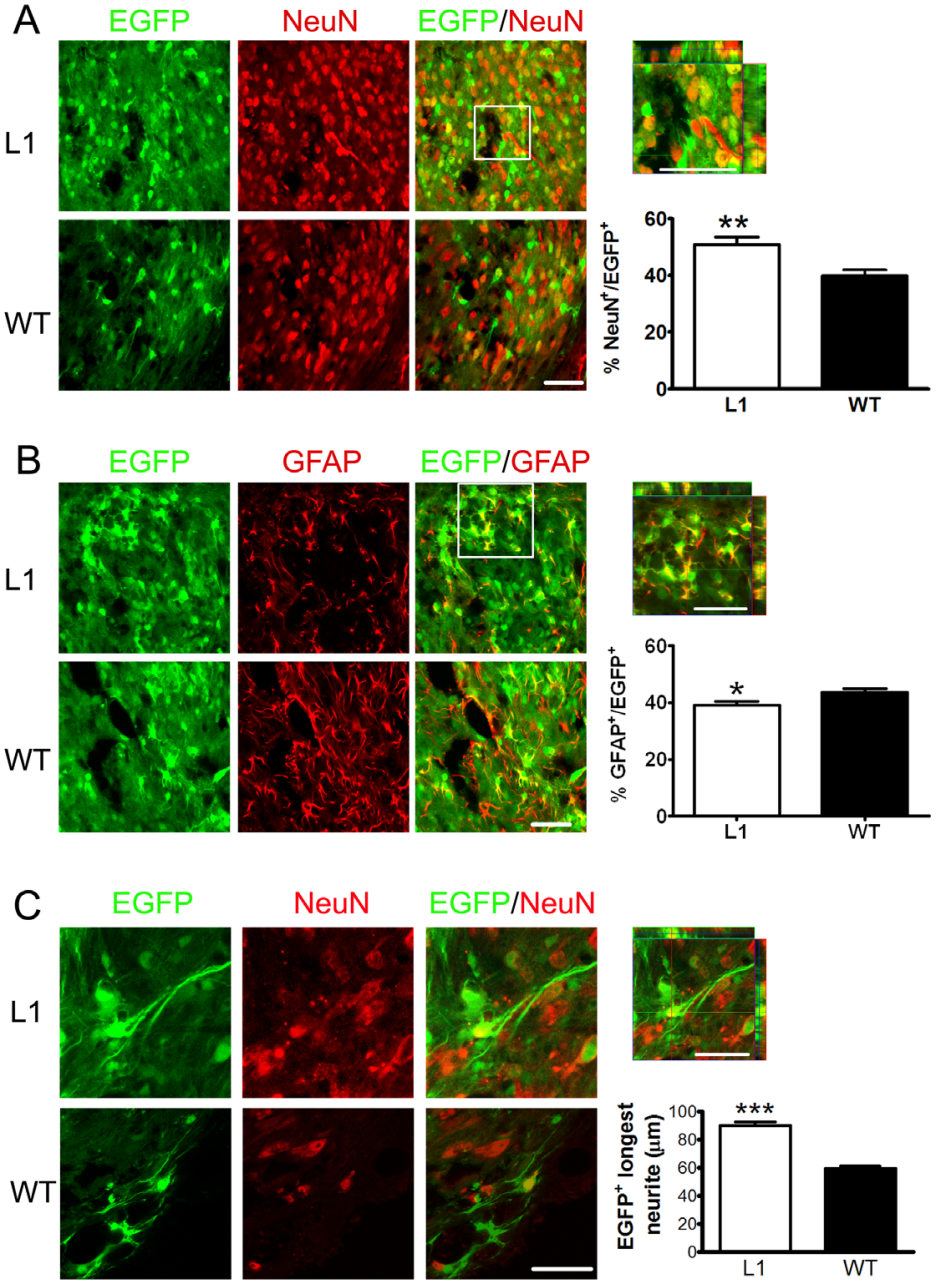
L1 overexpressing SENAs show increased neuronal differentiation and neurite outgrowth and decreased astrocytic differentiation after transplantation into the lesioned spinal cord when compared to wild-type SENAs. (**A**) Confocal images of L1 overexpressing and wild-type (WT) SENA grafts (green) immunostained with antibodies against the neuronal marker protein neuronal nuclear antigen (NeuN, red) six weeks after transplantation into the spinal cord. Scale bar, 50 µm. In the upper right, a Z-stack of 15 images of 1 µm thickness of the area outlined by a square in the merged image of the L1 overexpressing graft is shown with orthogonal views of the xz- and yz-planes showing EGFP-positive/NeuN-positive neurons. Scale bar, 50 µm. Percentages of NeuN-positive cells of all EGFP-positive cells six weeks after transplantation of L1 overexpressing (n = 6) and WT (n = 6) SENAs into the lesioned spinal cord (mean ± SEM) are shown. Student's t-test was performed for statistical analysis (** *p*<0.01). (**B**) Confocal images of L1 overexpressing and WT SENAs (green) immunostained with an antibody against GFAP (red) six weeks after transplantation. Scale bar, 50 µm. In the upper right, a Z-stack of 15 images of 1 µm thickness of the area outlined by a square in the merged image of the L1 overexpressing graft is shown with orthogonal views of the xz- and yz-planes showing EGFP-positive/GFAP-positive astrocytes. Scale bar, 50 µm. Percentages of GFAP-positive cells of all EGFP-positive cells six weeks after transplantation of L1 overexpressing (n = 6) and WT (n = 6) SENAs into the lesioned spinal cord (mean values ± SEM) are shown. Student's t-test was performed for statistical analysis (*p<0.05). (**C**) Laser scanning images of neurites from transplanted L1 overexpressing and WT SENAs (green) immunostained with antibodies against the neuronal marker NeuN (red) six weeks after transplantation. Scale bar, 50 µm. In the upper right, a Z-stack of 10 images of 1 µm thickness the L1 overexpressing graft is shown with orthogonal views of the xz- and yz-planes showing EGFP-positive/NeuN-positive neurons. Scale bar, 50 µm. The length of the longest neurite from neurons in the grafted SENAs was analyzed (L1, n = 7; WT, n = 7). Mean values ± SEM are shown. Student's t-test was performed for statistical analysis (*** *p*<0.001).

The percentage of neuronal nuclear antigen (NeuN)-positive neurons among the transplanted cells (Fig. 5A) was enhanced in L1 overexpressing SENAs when compared to wild-type SENAs six weeks after transplantation into the lesioned spinal cord, while GFAP-positive astrocytes were less abundant in L1 overexpressing SENAs (Fig. 5B) six weeks after transplantation. The percentage of oligodendrocytes was negligible in both groups and amounted to less than 1% of all EGFP-positive cells (not shown). Furthermore, the length of neurites of graft-derived NeuN-positive neurons was determined. The analysis revealed that neurites of graft-derived neurons were longer in L1 overexpressing versus wild-type SENAs (Fig. 5C). Thus, L1 ovexpressing SENAs favor neuronal differentiation, reduce astrocytic differentiation, and show increased neurite outgrowth of graft-derived neurons.

### L1 overexpression in SENAs shows beneficial effects on endogenous motoneurons and interneurons caudal to the lesion site

**Figure 6:**
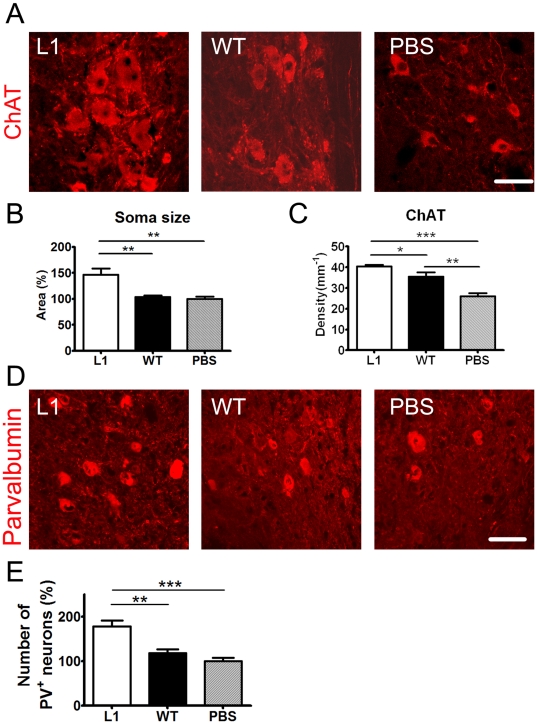
L1 overexpressing SENAs increase the soma size of host ChAT-positive motoneurons, the linear densities of ChAT-positive boutons on host motoneurons, and the number of host parvalbumin-positive interneurons in the lumbar spinal cord six weeks after transplantation. (**A**) Confocal images of host motoneurons, which were immunostained with an antibody against choline acetyltransferase (ChAT, red), caudal to the lesion site six weeks after transplantation. Scale bar, 50 µm. The relative soma size of host ChAT-positive motoneurons (**B**) and linear density of ChAT-positive puncta (**C**) caudal to the lesion site are shown (mean values ± SEM; L1 n = 6; wild-type (WT), n = 6; PBS, n = 5). One-way ANOVA with Tukey's *post hoc* test was performed for statistical evaluation (*, **, *** *p*<0.05, 0.01, 0.001). (**D**) Confocal images of host parvalbumin (PV)-positive neurons immunostained with an antibody against parvalbumin (red), caudal to the lesion site six weeks after transplantation. Scale bar, 50 µm (**e**) The number of PV-positive neurons in the lumbar spinal cord of mice grafted with L1 overexpressing SENAs (n = 6) and wild-type SENAs (n = 6) was compared to the number in sham-injected animals (n = 5) which was adjusted to 100%. One-way ANOVA with Tukey's *post hoc* test was performed for statistical evaluation (**, *** *p*<0.01, 0.001).

To analyze whether L1 overexpressing SENAs rescue imperiled host cells after spinal cord injury, mean areas of cell bodies of motoneurons and densities of synaptic terminals around the cell bodies of motoneurons caudal to the lesion site were measured six weeks after transplantation. Choline acetyltransferase (ChAT)-positive boutons have been shown to represent C-type synapses on motoneurons associated with muscarinic receptor type 2 [23], [24]. The linear density (number per unit length) of large perisomatic ChAT-positive boutons was increased in mice grafted with L1 overexpressing SENAs compared to mice grafted with wild-type SENAs or sham-injected with PBS (Fig. 6A, C). Mice grafted with wild-type SENAs also showed an enhanced linear density of ChAT-positive boutons caudal to the lesion site compared to sham-injected mice (Fig. 6C). In addition, the soma size of motoneurons was enhanced in mice that had been transplanted with L1 overexpressing SENAs six weeks after transplantation into the lesioned spinal cord when compared to wild-type SENAs and sham-injected mice (Fig. 6A, B). Furthermore, L1 overexpressing SENAs increased the number of parvalbumin-positive neurons caudal to the lesion site when compared to wild-type SENAs and sham-injected PBS (Fig. 6D, E). Thus, L1 overexpressing SENAs rescue endogenous motoneurons and interneurons distal to the lesion site after transplantation into the lesioned spinal cord.

### L1 overexpression in SENAs enhances catecholaminergic innervation distal to the lesion site in the lesioned spinal cord

**Figure 7:**
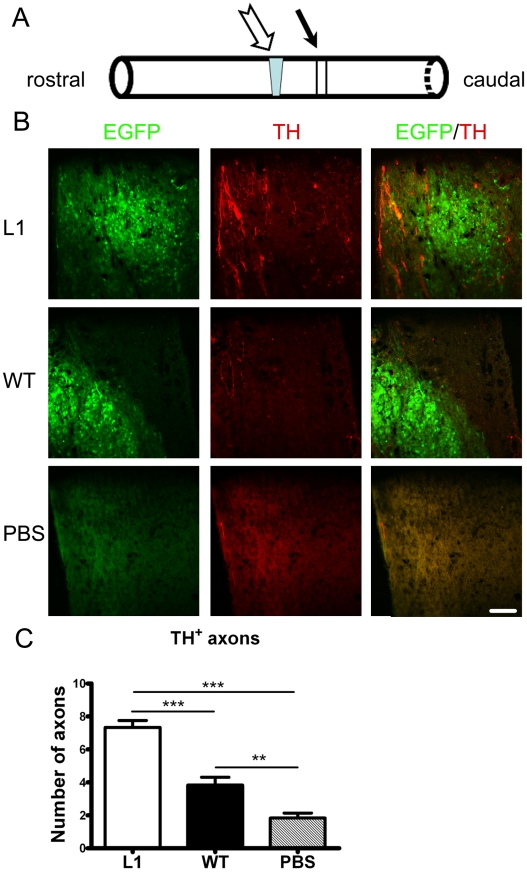
L1 overexpressing SENAs enhance the number of host tyrosine hydroxylase-positive axons caudal to the lesion site six weeks after transplantation. (**A**) Tyrosin hydroxylase (TH)-positive fibers crossing an arbitrary border 250 µm (indicated by a black arrow) caudal to the lesion site (indicated by a hollow arrow) were quantified six weeks after transplantation. (**B**) Laser scanning images of TH-positive (red) axons 250 µm caudal to the lesion site in spinal cords grafted with L1 overexpressing SENAs (n = 6), wild-type (WT) SENAs (n = 6), or PBS (n = 6). (**C**) The number of TH-positive axons per section 250 µm caudal to the lesion site is shown. (mean values ± SEM) One-way ANOVA with Tukey's *post hoc* test was performed for statistical evaluation (**, *** *p*<0.01, 0.001).

Monoaminergic descending pathways, located in the ventral and lateral columns of the spinal cord [25], [26] modulate the excitability of spinal cord circuitries capable of initiating and controlling rhythmic coordinated movements [27], [28]. To assess whether transplantation of L1 overexpression in SENAs could enhance monoaminergic reinnervation of the spinal cord caudal to the lesion site, the numbers of catecholaminergic (tyrosine hydroxylase (TH)-positive) and serotonergic (serotonin (5-HT) transporter-positive) axons caudal to the lesion site were determined six weeks after transplantation (Fig. 7A). L1 overexpressing SENAs showed higher numbers of catecholaminergic axons distal to the lesion site when compared to mice grafted with wild-type SENAs or sham-injected with PBS (Fig. 7B, C). Analysis of the numbers of serotonergic axons distal to the lesion site revealed a tendency towards increased numbers in the L1 overexpressing SENAs versus wild-type SENAs and the PBS injected group, which was, however, statistically not significant (Fig. S3).

## Discussion

In this study we show that SENAs, which are neural stem cell-derived aggregates consisting mainly of radial glial cells and neurons, overexpressing neural cell adhesion molecule L1 enhance functional recovery after early syngeneic transplantation into the lesioned murine spinal cord. Morphological analysis of transplanted cells revealed that overexpression of L1 beneficially influences survival and migration of grafted SENAs, neuronal differentiation of and neurite outgrowth from grafted SENAs. Furthermore, L1 overexpressing SENAs led to increased numbers of perisomatic synapses and soma size of endogenous motoneurons distal to the lesion site and rescued imperiled endogenous parvalbumin-positive interneurons.

A previous study [5] showed that L1 overexpressing embryonic stem (ES) cells transplanted as single cell suspension had beneficial effects on the survival of ES cells after transplantation into the lesioned murine spinal cord. As survival of grafted cells was poor in this former study with only a minority of wild-type ES cells surviving four weeks after transplantation, we used the SENA differentiation protocol to further enhance survival of grafted L1 overexpressing ES cells and to monitor the effects of the combined therapy on locomotor functional recovery that was not measured in the previous study. In our preliminary study cellular and molecular parameters were not analyzed at all – except for measuring the regrowth of the corticospinal tract which is not the decisive determinant in locomotor recovery with a T8–10 lesion site. We now studied the tracts involved in locomotor recovery and the synaptic coverage of motoneurons and other parameters that reflect the improvement in functional regeneration. In agreement with the previous study [5], we found enhanced survival of transplanted L1 overexpressing SENAs when compared to wild-type SENAs. In contrast to the previous study [5], where wild-type ES cells did not survive four weeks after transplantation, wild-type SENAs showed robust survival up to six weeks after transplantation in the present study, indicating beneficial effects of the SENA differentiation protocol. The enhanced survival of transplanted cells may be due to the fact that SENAs that are transplanted as aggregates create an environment that enhances survival of grafted cells under the unfavorable conditions within the lesioned spinal cord, while ES cells – when differentiated by the five stage protocol [20] and transplanted as a single cell suspension - are less capable to create a beneficial tissue environment in the absence of L1. L1 exerts its beneficial effects via homophilic and heterophilic interactions with the cell surfaces of neighbouring cells or the extracellular matrix [10], [11], [12], [14]. Also, L1 can be shed from the cell surface by proteolysis at distinct sites in the extracellular domain [29], [30], [31], thus releasing L1 into the extracellular space to act back on the L1 expressing cells in an autocrine fashion and in a paracrine fashion on cells that are reached within its diffusion radius. The enhanced survival of grafted cells in L1 overexpressing versus wild-type cells is not due to an influence of L1 on proliferation because the numbers of proliferating cells amounted to less than 1% in both groups. Interestingly, L1 increased graft survival caudal but not rostral to the lesion site by mechanisms yet unknown. However, it has been shown that in the injured spinal cord more proinflammatory molecules (e.g. TNFα, IL-β) and less metabolism-related genes are differentially expressed caudal than rostral to the lesion-site [32], [33], indicating a more hostile environment caudal than rostral to the lesion site [34]. Thus, the beneficial effects of L1 appear more prominent in an environment less favorable to survival.

The determination of an optimal time point for stem cell transplantation after spinal cord injury is important to ensure survival of transplanted ES cells. Due to the environment in the acutely injured spinal cord, one to two weeks after injury were previously regarded as the optimal time period for transplantation [6], [7], [8], [35], [36], [37], [38] since immediately after spinal cord injury, inflammatory cytokines are expressed at the lesion site that can lead to apoptosis of grafted cells or preferential differentiation into astrocytes. Thus, few studies address time points of cell transplantation early after spinal cord injury. Hofstetter et al. [39] found that mesenchymal stem cells rarely survived transplantation immediately after spinal cord injury and did not improve locomotor function. Ogawa et al. [37] reported that only few neural stem/progenitor cells survived when transplanted 24 h after spinal cord injury. In the present study, we show robust survival of ES cells after transplantation three days after spinal cord injury leading to enhanced locomotor functional recovery, emphasizing the beneficial effect of the combination of the SENA differentiation protocol and overexpression of L1. While in our previous study [5] only L1 overexpressing ES cells transplanted as single cell suspension showed significant survival after transplantation seven days after spinal cord injury, even wild-type ES cells, as shown in the present study, survived transplantation as SENAs three days after spinal cord injury in the present study.

Transplantation of L1 overexpressing SENAs reduced scar formation in the compression-lesioned spinal cord. After spinal cord injury, formation of the scar by extracellular matrix molecules and glial cells is thought to be a major factor limiting recovery [40]. This view is supported by studies showing that reduced scar formation is accompanied by improved functional recovery [21], [28], [41] after spinal cord injury. In our study, the scar volume was significantly reduced in animals transplanted with L1 overexpressing SENAs when compared to sham-injected mice. This observation is in accordance with the finding of Chen et al. [17] who observed that virus-mediated expression of L1 in the lesioned spinal cord decreased the scar volume and expression of GFAP.

Furthermore, L1 overexpressing SENAs slightly decreased the microglial/macrophage reaction both rostral and caudal to the lesion site when compared to mice injected with wild-type SENAs or PBS. The reason why the number of these cells was decreased in the vicinity of L1 overexpressing versus wild-type grafts is currently not understood, but reduction of these cells may contribute to enhanced numbers of L1 overexpressing SENAs. However, this view is contentious since previous studies showed that microglial cells have the potential to both increase and decrease neuronal survival [42].

In the present study, L1 influenced the host environment not only by reducing the scar volume, but also increased neuronal differentiation and neurite outgrowth and decreased astrocytic differentiation of grafted SENAs. This is in line with previous results reporting an enhanced neuronal differentiation and decreased astrocytic differentiation of L1 overexpressing embryonic stem cell-derived neural precursor cells and L1 overexpressing SENAs after transplantation into the quinolinic acid-lesioned striatum [13] and the brain of Parkinsonian mice [16], respectively. This phenomenon is probably due to an instructive role of L1 by heterophilic and homophilic mechanisms in the lineage decision since *in vitro* L1 enhances neuronal differentiation and decreases astrocytic differentiation of multipotent and neuron/astrocyte-restricted neural precursor cells [14]. Furthermore, L1 has been shown to mediate neuronal survival and neurite outgrowth *in vitro* by interaction with ankyrin, members of the ERM (ezrin, radixin, and moesin) family, actin stress fibers, and the AP-2 adaptor complex (for review see [43]). These mechanisms are likely to play an important functional role also *in vivo* in the present lesion paradigm.

These interactions with the cytoskeleton are likely the basis for the enhanced migration of grafted cells into the host tissue, another beneficial property of L1 that has previously been described [13], [16]. Also, L1 overexpressing SENAs enhanced the regrowth of TH-positive fibers distal to the lesion site. Homophilic or heterophilic mechanisms have been shown to enhance axonal out-/re-growth of L1 expressing axons which can form L1-mediated fascicles which support each others growth. L1 expressing dopaminergic descending axons, originating in the substantia nigra, tegmentum, and reticular formation, are located in the ventral and lateral columns of the spinal cord [44]. They mediate the excitability of spinal cord circuitries [27], which correlates with locomotor functional recovery in the injured spinal cord of mice [28]. L1 may help these neurons to regrow by using this molecule as a conducive homophilic interaction partner between the non-myelinated L1 expressing dopaminergic axons. Furthermore, L1 has also been shown to promote survival of fetal dopaminergic neurons *in vitro* [45]. Thus, it is not surprising that L1 overexpressing SENAs enhanced dopaminergic reinnervation in the spinal cord caudal to the lesion site, thereby contributing to functional recovery.

After spinal cord injury, death of motoneurons, especially in the lumbar enlargements, can contribute to locomotor dysfunction [46]. An important aim of stem cell transplantation thus is to rescue endogenous motoneurons after spinal cord injury. In the present study, L1 overexpressing SENAs rescued host motoneurons as indicated by an enlarged soma size of motoneurons distal to the lesion site while wild-type SENAs did not. Furthermore, the density of cholinergic puncta on motoneurons was higher in mice transplanted with L1 overexpressing SENAs indicating enhanced numbers of cholinergic perisomatic synapses. Since in the intact spinal cord, cholinergic perisomatic synapses regulate motoneuron excitability during locomotion [47], L1 may promote recovery after spinal cord injury by rescuing motoneurons by increased cholinergic innervation of these cells. L1 has previously been shown to be a survival factor for motoneurons *in vitro* presumably by exerting L1-triggered survival promoting functions in these cells via activating several signaling mechanisms, including the MEK and PI3K pathways and regulation of Ca^++^ influx, in addition to interacting with cytokine receptors [48], [49], thus rescuing endogenous motoneurons by direct homophilic and heterophilic L1 interactions between pre- and post-synaptic neuronal cell surfaces.

Not only motoneurons, but also interneurons that express parvalbumin are rescued by either homophilic or heterophilic interactions with the host tissue from cell death by L1 overexpressing SENAs. Interneurons in the lumbar spinal cord influence the pattern of motor activity generated within the spinal cord [50].L1 overexpressing SENAs increased the number of parvalbumin-positive interneurons distal to the lesion site when compared to wild-type SENAs. This rescue of parvalbumin-positive interneurons by L1 overexpressing SENAs is also likely to contribute to the positive effects on endogenous motoneurons and locomotor recovery. Because parvalbumin has a high affinity for intracellular calcium, it can reduce the hypopolar refractory period after an action potential, allowing the neuron to recover quickly and fire at a higher frequency [51], [52].

Using a set of behavioral analyses to observe different locomotor abilities, mice transplanted with L1 overexpressing SENAs showed enhanced locomotor recovery when compared to wild-type SENAs. Three weeks after transplantation, mice grafted with L1 overexpressing SENAs showed a higher BBB score and improved foot-stepping angle when compared to wild-type SENAs. This improvement was seen also six weeks after transplantation. The foot-stepping angle and the BBB score are parameters assessing plantar stepping ability [21], [28], [53], requiring a low degree of supraspinal control. The rump-height index and the extension–flexion ratio, which require more complex motor control and coordination compared with plantar stepping, were not improved by L1 overexpressing SENAs when compared to wild-type SENAs although L1 overexpressing SENAs but not wild-type SENAs showed an enhanced rump-height index when compared to sham-inject mice six weeks after transplantation. It is thus conceivable that transplantation of L1 overexpressing SENAs influences local adaptive responses after injury. Morphological analysis supports this hypothesis as transplantation of L1 overexpressing SENAs increased numbers of dopaminergic fibers, cholinergic synapses on spinal motoneurons, and soma size of motoneurons distal to the lesion site. These observations are in agreement with previous studies showing that functional recovery after spinal cord injury in mice strongly correlates with the degree of monoaminergic innervation of the distal spinal cord and with cholinergic perisomatic innervation of motoneurons [28].

These cellular parameters indicating a beneficial influence of L1 overexpressing SENAs after injury are supported by electrophysiological measurements of the Hoffmann reflex (H-reflex), which represents an electrically elicited analog of the spinal stretch reflex providing information about the functional properties of Ia afferents and homonymous alpha-motoneurons under physiological and pathological conditions [54]. Lee et al. [22] demonstrated that the H-reflex is a useful tool for the assessment of motoneuron pool excitability in spinal cord-injured mice and observed that the increase of H/M ratios under alternating stimulation conditions correlates with locomotor recovery. The H/M ratios were increased at lower frequencies (0.1–0.5 Hz) in mice transplanted with L1 overexpressing SENAs when compared to mice grafted with wild-type SENAs or sham-injected with PBS, further supporting the notion that L1 overexpressing SENAs are beneficial for functional recovery after spinal cord injury. The mechanism underlying the dependence of the increase of the H/M ratio on the stimulation frequency is currently not understood.

In summary, the differentiation of mouse ES cells into SENAs combined with overexpression of the neural cell adhesion molecule L1 led to enhanced neuronal differentiation and migration, as well as neurite outgrowth, and better survival of grafted cells after early transplantation into the lesioned spinal cord with a reduced scar volume when compared to wild-type SENAs or sham-injected PBS. L1 overexpressing SENAs enhanced monoaminergic reinnervation distal to the lesion-site, increased cholinergic synaptic reinnervation of endogenous motoneurons, and rescued host motoneurons. This led to increased and sustained functional recovery. These observations encourage, in addition to favoring the genetic manipulation with a beneficial adhesion molecule, the transplantation of embryonic stem cells early after spinal cord injury. L1 expression in the microenvironment of the lesioned spinal cord is a novel finding in its functions that would make it more attractive for pre-clinical studies in spinal cord regeneration and most likely other diseases of the nervous system. The recent observation that adhesion molecules are not only important determinants for the molecular, but also mechanically guided lineage selection and differentiation of stem cells [55] adds to the concept that adhesion molecules are essential for stem cell biology. In this respect the study of L1 in several conditions of applications is a prerequisite for further steps in therapeutic applications.

## Materials and Methods

### Generation and isolation of L1 overexpressing SENAs

The generation of ES cells overexpressing full length L1 under the control of the promoter of the isoform 1 of the 3-phosphoglycerokinase and expressing EGFP under the control of the chick β-actin promoter has been previously described [13]. Culture of undifferentiated L1 overexpressing and non-engineered, wild-type ES cells and differentiation into SENAs was carried out as described [19]. After propagation of ES cells on mitomycin C (Roth, Karlsruhe, Germany)-inactivated mouse embryonic fibroblasts (stage 1), embryoid bodies were formed via hanging drops consisting of 800 cells per 20 µl drop for two days and were kept for an additional two days in bacterial Petri dishes (stage 2). Selection of nestin-positive cells was performed in serum-free medium containing 5 µg/ml insulin, 50 µg/ml transferrin, 30 nM selenium chloride, and 5 µg/ml fibronectin (all from Sigma, St. Louis, MO, USA) (stage 3). Selected nestin-positive cells were seeded onto poly-L-ornithine-coated cell culture dishes and maintained for four weeks under the influence of fibroblast growth factor-2 (FGF-2; 20 ng/ml; PreproTech, Rocky Hill, NY, USA) (prolonged stage 4). For differentiation, FGF-2 was then withdrawn for seven days to induce terminal differentiation of SENA-derived young neurons (stage 5).

To obtain highly enriched SENA preparations for transplantation, cultures were treated with 0.3 µg/ml collagenase XI (Sigma) at 37°C for 10 min, thus gently detaching entire SENAs comprising aggregates of radial glial cells and neurons from the monolayer of neural precursor cells surrounding the aggregates. Detached SENAs were carefully pipetted up and down 10 times with a fire-polished Pasteur pipette to separate monolayer cells from SENAs which remained intact during this procedure. SENAs were harvested by sedimentation at 1×g for 2 minutes and resuspended in phosphate-buffered saline, pH 7.4 (PBS) to pick SENAs individually using a 10 µl pipette tip. For transplantation, a suspension of SENAs was adjusted to 10 SENAs/µl. Cell number per SENAs was estimated by dissociation with trypsin, showing that, on average, one SENA consisted of 10,000 cells.

### Transplantation

Three days before transplantation, the spinal cords of two-month-old female C57BL/6J mice were lesioned. Laminectomy was performed at the T8–T10 level with mouse laminectomy forceps (Fine Science Tools, Heidelberg, Germany). A mouse spinal cord compression device was used to elicit a compression injury [56]. Compression force (degree of closure of the forceps) and duration were controlled by an electromagnetic device. The spinal cord was maximally compressed (100%, according to the operational definition of Curtis and colleagues [56]) for 1 s by a time-controlled current flow through the electromagnetic device. On the day of transplantation, SENAs were harvested and resuspended in PBS with ten SENAs (approximately 10,000 cells each) per µl. One µl of L1 overexpressing SENAs, wild-type SENAs, or PBS only was injected 0.5 mm both rostral and caudal to the lesion site using a glass micropipette (tip diameter 100 µm) 1 mm deep into the spinal cord. Grafts were analyzed six weeks after transplantation. All animal experiments were approved by the University and State of Hamburg Animal Care Committees (permit number: G18/07). Transplantation and subsequent behavioral, electrophysiological, and histological analyses were performed in a blinded manner.

### Evaluation of locomotor behavior

The recovery of ground locomotion was evaluated using the Basso, Beattie, Bresnahan (BBB) rating scale [57] modified for mice [58], and a single-frame motion analysis [21]. The latter method includes evaluation of three parameters in two different tests: a beam walking test (foot-stepping angle and rump-height index) and a test to assess voluntary movements without body weight support (extension–flexion ratio). All mice received a spinal cord compression lesion as described above. Mice were then randomly assigned to three groups that received an intraspinal injection of L1 overexpressing SENAs (n = 14), wild-type SENAs (n = 14), or PBS only (n = 12). In the L1 overexpressing SENAs group and the PBS group one animal died during surgery. Assessments were performed before and at one, three, and six weeks after the injury. Values for the left and right extremities were averaged. Only animals that showed a foot-stepping angle of 160° or more one week after injury were considered severely lesioned. All other animals were excluded from behavioral and morphologic analysis. In the L1 overexpressing SENA group 2 animals were excluded, in the wild-type SENA group 2 animals were excluded. In the PBS only group 1 animal was excluded. Furthermore, 1 animal in the L1 overexpressing SENA group, 2 animals in the wild-type SENA group, and 2 animals in the PBS group died in the course of the experiments and were thus excluded. Recovery indices were used as a measure of functional recovery for each individual animal as previously described [21]. The recovery index (RI) is an individual animal estimate for any given parameter described above and is calculated in percent as: RI = [(X_7+n_−X_7_)/(X_0_−X_7_)]×100, where X_0_, X_7_ and X_7+n_ are experimental values prior to operation, 7 days after injury, and a time-point n days after spinal cord injury, respectively. One-way ANOVA with Tukey's *post hoc* test was performed for statistical evaluation.

### Rate depression of the H-reflex electromyography (EMG) recordings

The EMG recordings were performed as described [22]. The sciatic nerve was stimulated using bipolar electrical pulses of 0.2 ms duration to elicit reflex responses. The stimulus intensity was gradually increased until both M- and H-waves with latencies of approximately 2 and 5 ms, respectively, were visible. After the threshold measurement, the stimulus intensity was further increased until maximal and stable H-responses were elicited. Thereafter, stimulation continued at the defined suprathreshold level at frequencies of 0.1, 0.3, 0.5, 1, 2, 10, 20 Hz. Six consecutive responses were recorded at each frequency. The amplitudes of M- and H-waves were measured as peak-to-peak values, averaged (excluding the first response at each frequency) and used to calculate H/M ratios. The latencies of the responses were measured as time elapsed between the trigger and peak of each waveform.

### Scar volume

Six weeks after transplantation, mice were sacrificed and spinal cords were cut in sagittal sections (20 µm thick) on a cryostat. Serial sections spaced 200 µm apart were stained with antibodies against GFAP (Dako, Carpinteria, CA, USA) and fibronectin (Sigma) to estimate the scar volume using the Cavalieri principle.

### Graft volume and cell density in the graft

Unbiased estimates of the total number of grafted cells and graft volume per animal were calculated six weeks after transplantation according to the optical disector and Cavalieri methods. An Axioskop microscope (Carl Zeiss Microimaging, Jena, Germany) equipped with a motorized stage and a Neurolucida software-controlled computer system was used for quantitative analysis (MicroBrightField Europe, Magdeburg, Germany). Graft volume and cell density of the graft were determined measuring every tenth section of the graft. Transplanted cells were identified by their EGFP signal. Graft areas were outlined on digitized images to calculate volumes based on section thickness and frequency. Using random sampling in the graft core and in the periphery of the graft, cell counts were performed according to the optical disector principle at a magnification of ×40. Nuclei of DAPI-positive and EGFP-positive grafted cells were counted according to their position in each disector. For statistical evaluation, Student's t-test was used.

### Migration of transplanted cells

The graft edge was outlined at low magnification (×5) in digitized images. The shortest distances of 400 individual cells from the graft edge of recipient animals were determined at higher magnification (×40). An Axioskop microscope (Carl Zeiss Microimaging) equipped with a motorized stage and a Neurolucida software-controlled computer system was used for quantitative analysis (MicroBrightField Europe, Magdeburg, Germany).

### Immunohistochemistry

For immunohistochemical analysis, mice were perfused with 4% formaldehyde and 0.1% CaCl2 in 0.1 M cacodylate buffer, pH 7.3. Perfused spinal cord tissue was soaked in 20% sucrose overnight, frozen in liquid nitrogen-cooled 2-methyl-butane, and cut on a cryostat at 20 µm. Primary antibodies, used at 4°C overnight, were monoclonal mouse antibodies against CNPase (1∶1000; Sigma), NeuN (1∶1000; Millipore, Hofheim, Germany), parvalbumin (1∶1000; Sigma); polyclonal rabbit antibodies against GFAP (1∶1000; Dako), TH (1∶800; Millipore), and Iba-1 (1∶1500; Wako Chemicals, Richmond, VA, USA); polyclonal goat antibody against ChAT (1∶100; Millipore); polyclonal guinea pig antibody against 5-HT transporter (1∶1000; Millipore). For detection of primary antibodies, appropriate secondary antibodies, coupled to Cy2 or Cy3 (all from Dianova, Hamburg, Germany), were used. Specimens were examined with a confocal laser-scanning microscope (LSM510; Carl Zeiss Microimaging).

### Differentiation of SENAs *in vivo*

o determine total numbers of donor cells *in vivo*, EGFP-positive cells were counted and the ratio of cell type-specific or functional marker-positive cells of all EGFP-positive cells was calculated. At least three independent experiments in duplicates and at least 1000 cells per marker and experiment were analyzed. Percentages of double-labeled cells were determined and mean values ± standard error of the mean (SEM) were calculated. Student's t-test was used for statistical evaluation.

### Quantification of neurite outgrowth from neurons derived from grafted cells

To determine the maturation of neurons differentiated from transplanted SENAs, the longest neurite length per cell was measured using the Neurolucida software (MicroBrightField Europe). Sections were stained with an anti-NeuN antibody. In each experiment, at least 50 cells per section were analyzed. For statistical evaluation Student's t-test was used.

### Quantification of glial and microglial/macrophage reactions of the host tissue to the graft

Quantification of glial and microglial/macropahge reactions was performed as described [16]. To determine the reaction of the host tissue to grafted cells, immunostaining with antibodies against GFAP to analyze astrogliosis and Iba-1 to analyze microglial/macrophage activation was performed. All sections were stained at the same batch. Confocal images were taken in the vicinity of the edge of the graft in mice transplanted with L1 overexpressing and wild type SENAs, and corresponding areas in mice sham-injected with PBS. At least 20 images were analyzed in each mouse. The software Image J (http://rsbweb.nih.gov/ij/index.html) was used to measure fluorescence intensities from all 3 groups. Fluorescence intensities in the L1 overexpressing and wild type SENA group were normalized to the PBS group. For statistical evaluation one-way ANOVA followed by Tukey's post hoc test was used.

### Quantification of motoneuron soma size and perisomatic puncta

Estimations of soma size and perisomatic puncta were performed as described [53]. Longitudinal spinal cord sections stained for ChAT were examined under a fluorescence microscope to select sections that contained motoneuron cell bodies at least 500 µm distal to the lesion scar. Stacks of images of 1 µm thickness were obtained on a LSM 510 confocal microscope (Zeiss) using a 40× oil immersion objective lens and digital resolution of 1024×1024 pixel. Four adjacent stacks (frame size 115×115 µm) were obtained consecutively in a rostro-caudal direction in order to sample motoneurons. One image per cell at the level of the largest cross-sectional area was used to measure soma area, perimeter and number of perisomatic puncta. Motoneurons were identified by immunolabeling in sections stained for ChAT and by the size of the cell bodies (unstained profiles surrounded by immunoreactive puncta) and by bisbenzimide counterstained nuclei. Areas and perimeters were measured using the Image Tool 2.0 software program (University of Texas, San Antonio, TX, USA; free software available at http://ddsdx.uthscsa.edu/dig/). Linear density was calculated as the number of perisomatic puncta per unit length. For statistical evaluation, one-way ANOVA followed by Tukey's post hoc test was used.

### Quantification of parvalbumin-immunopositive interneurons in the lumbar spinal cord

To determine the alteration of parvalbumin-immunopositive interneurons in the lumbar spinal cord after transplantation, longitudinal spinal cord sections stained for parvalbumin were examined according to optical dissector and Cavalieri methods, respectively. An Axioskop microscope (Zeiss) equipped with a motorized stage and a Neurolucida software-controlled computer system was used for quantitative analysis (MicroBrightField Europe). Cell counts were performed according to the optical dissector (Cavalieri) principle at a magnification of 40. Total numbers of parvalbumin-immunopositive interneurons in the L1 overexpressing and wild-type SENA group were normalized to the PBS group. For statistical evaluation one-way ANOVA followed by Tukey's post hoc test was used.

### Quantification of monoaminergic innervation of the spinal cord distal to the lesion site

To assess monoaminergic innervation of the spinal cord caudal to the lesion, TH-immunopositive and 5-HT-transporter-immunopositive axons projecting beyond an arbitrarily selected border 250 µm caudal to the lesion site were counted in spaced serial parasagittal sections six weeks after injury.

## Supporting Information

Figure S1 **Proliferation of grafted cells is low six weeks after transplantation.** Confocal images of L1 overexpressing (L1+) and wild-type (WT) SENA grafts (green) immunostained with an antibody against Ki-67 (red), 6 weeks after transplantation. Less than 1% of grafted cells were Ki-67-positive in both groups. Scale bar = 50 µm.
(TIF)

Figure S2 **Grafted cells occasionally show close proximity to host neurons.** (**A**) Representative confocal images of host tissue surrounding L1 overexpressing grafts (green) immunostained with an antibody against NeuN (red). Scale bar, 10 µm. Note grafted cells projecting processes to host neurons (*, host neurons). (**B**) Representative confocal images of host tissues surrounding the graft (green) immunostained with an antibody against ChAT (red). Scale bar, 20 µm. Arrow heads indicate close proximity of Chat-positive synapses (red) and grafted cells (green).
(TIF)

Figure S3 **Serotonergic innervation caudal to the lesion site does not differ among the experimental groups.** Mean numbers of host serotonergic (5-HT-transporter-positive) fibers crossing an arbitrary border 250 µm caudal to the lesion site six weeks after transplantation are shown (mean values ± SEM). One-way ANOVA with Tukey's *post hoc* test was performed for statistical evaluation.
(TIF)
