# Supplementary figures and images for: Embryonic Stem Cell-Derived L1 Overexpressing Neural Aggregates Enhance Recovery after Spinal Cord Injury in Mice
Source: PLoS One. 2011 Mar 18;6(3):e17126. doi: 10.1371/journal.pone.0017126 (PMC3060805; doi:10.1371/journal.pone.0017126)

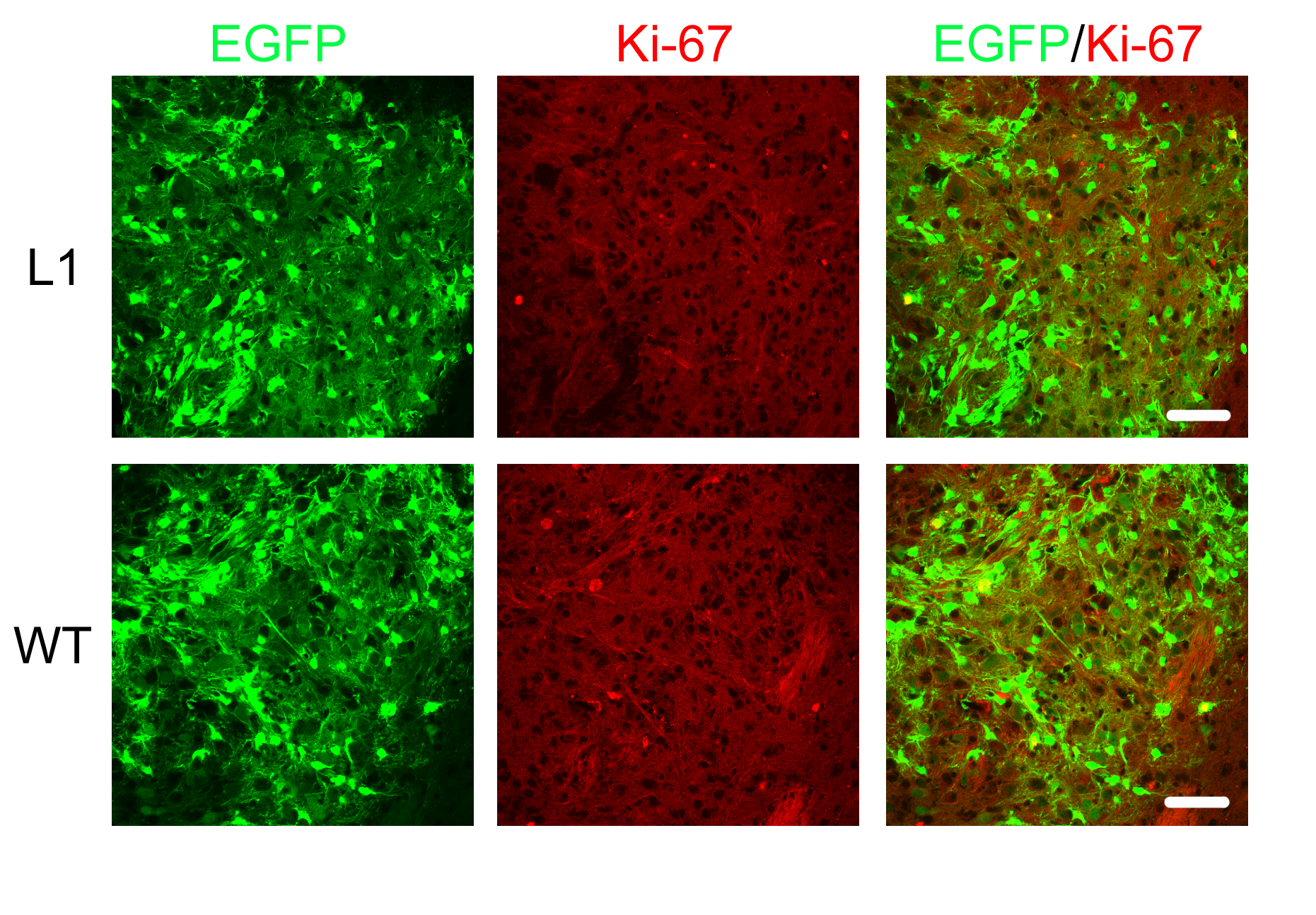

Supplement: Figure S1 — Proliferation of grafted cells is low six weeks after transplantation. Confocal images of L1 overexpressing (L1+) and wild-type (WT) SENA grafts (green) immunostained with an antibody against Ki-67 (red), 6 weeks after transplantation. Less than 1% of grafted cells were Ki-67-positive in both groups. Scale bar = 50 µm. (TIF) [file pone.0017126.s001.tif]

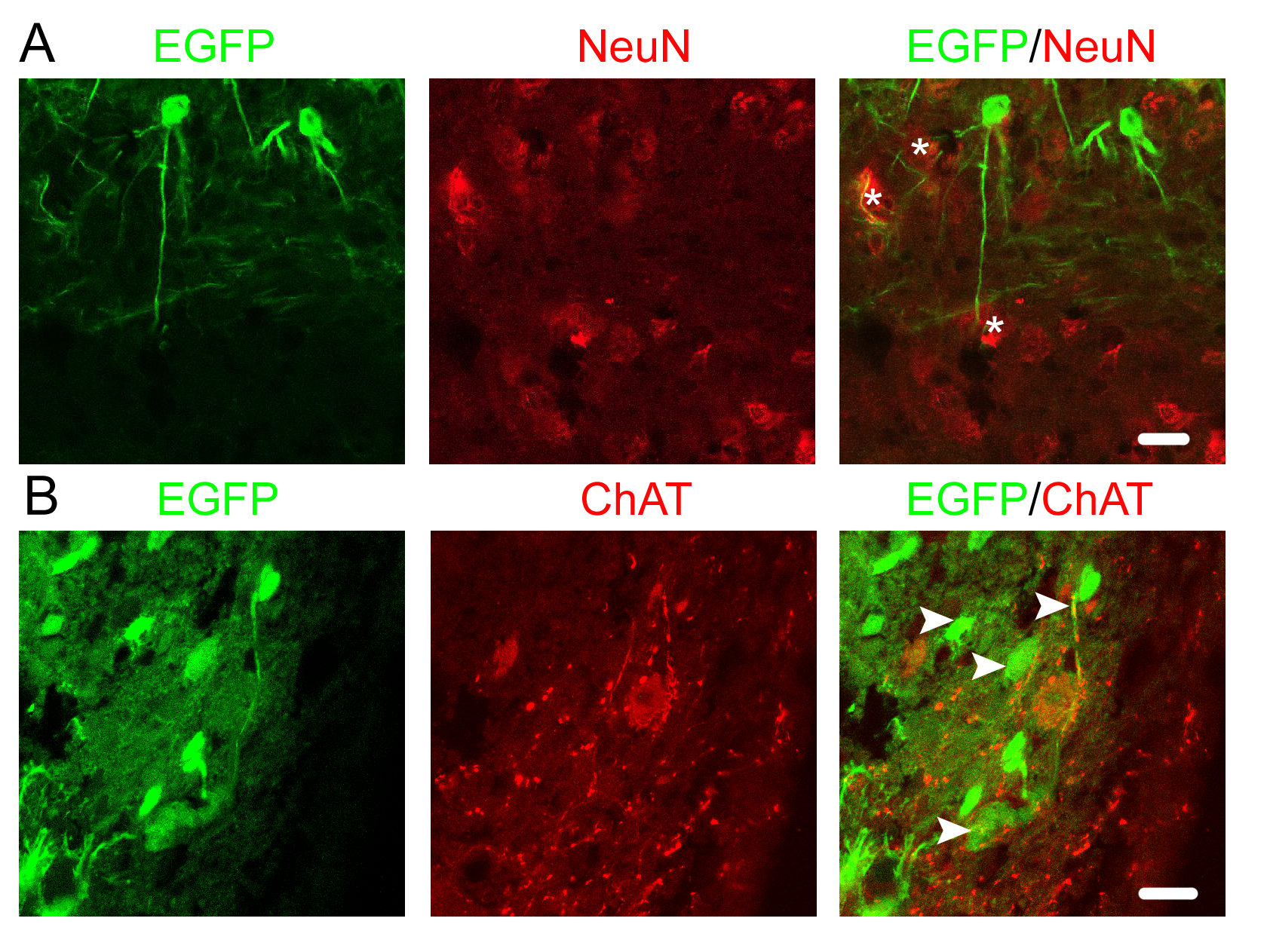

Supplement: Figure S2 — Grafted cells occasionally show close proximity to host neurons. (A) Representative confocal images of host tissue surrounding L1 overexpressing grafts (green) immunostained with an antibody against NeuN (red). Scale bar, 10 µm. Note grafted cells projecting processes to host neurons (*, host neurons). (B) Representative confocal images of host tissues surrounding the graft (green) immunostained with an antibody against ChAT (red). Scale bar, 20 µm. Arrow heads indicate close proximity of Chat-positive synapses (red) and grafted cells (green). (TIF) [file pone.0017126.s002.tif]

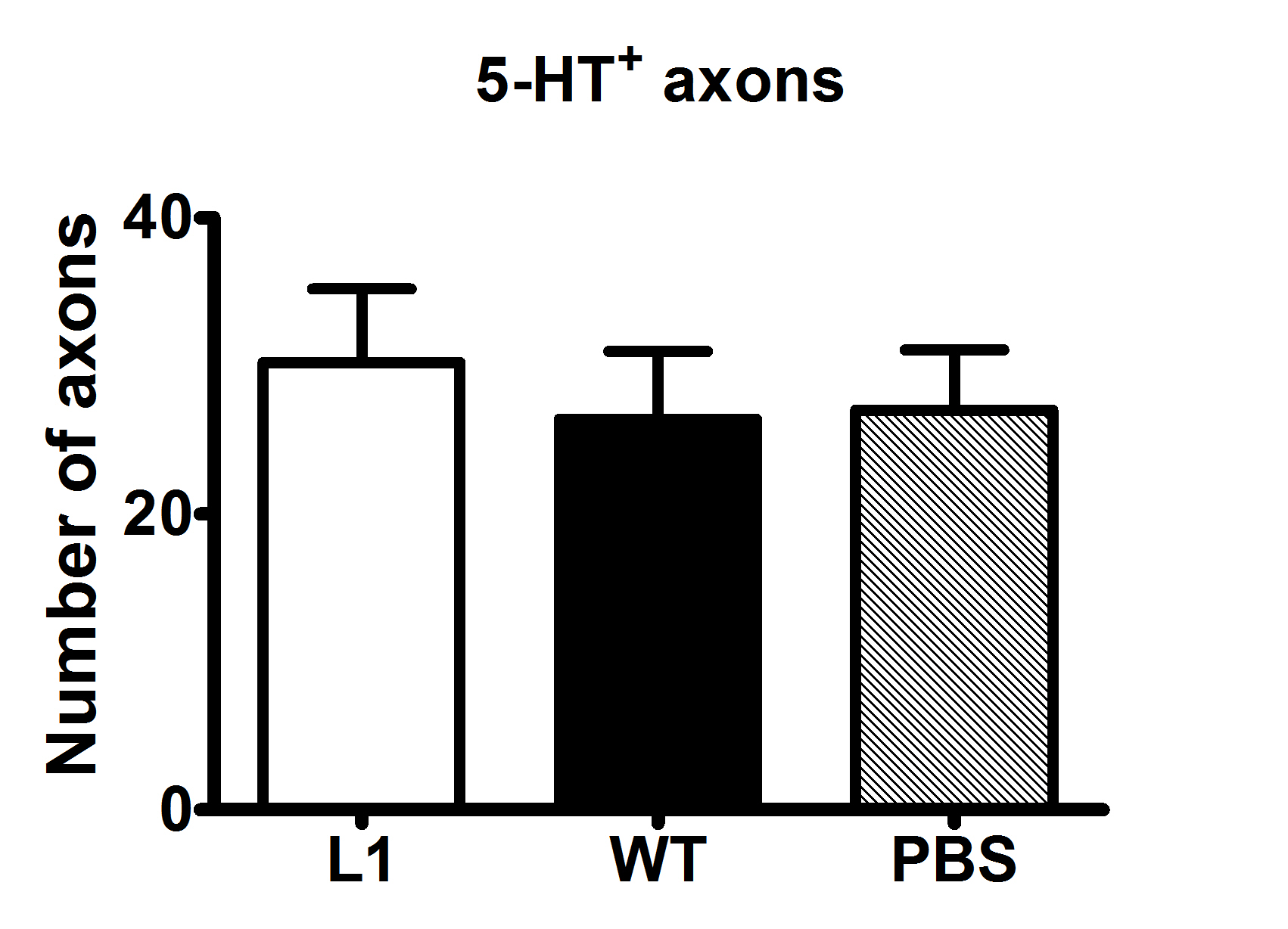

Supplement: Figure S3 — Serotonergic innervation caudal to the lesion site does not differ among the experimental groups. Mean numbers of host serotonergic (5-HT-transporter-positive) fibers crossing an arbitrary border 250 µm caudal to the lesion site six weeks after transplantation are shown (mean values ± SEM). One-way ANOVA with Tukey's post hoc test was performed for statistical evaluation. (TIF) [file pone.0017126.s003.tif]
